# Assessing Knowledge Sharing in Cancer Screening Among High-, Middle-, and Low-Income Countries: Insights From the International Cancer Screening Network

**DOI:** 10.1200/JGO.19.00202

**Published:** 2019-10-04

**Authors:** Douglas M. Puricelli Perin, Amanda L. Vogel, Stephen H. Taplin

**Affiliations:** ^1^ Frederick National Laboratory for Cancer Research, Frederick, MD; ^2^ Center for Global Health, National Cancer Institute, National Institutes of Health, Rockville, MD

## Abstract

**PURPOSE:**

As the global burden of cancer rises, global knowledge sharing of effective cancer control practices will be critical. The International Cancer Screening Network (ICSN) of the US National Cancer Institute facilitates knowledge sharing to advance cancer screening research and practice. Our analysis assessed perceptions of ICSN’s value and knowledge sharing in cancer screening among participants working in high-income countries (HICs) and low- and middle-income countries (LMICs).

**METHODS:**

In 2018, the National Cancer Institute fielded a self-administered, online survey to 665 ICSN participants from both HICs and LMICs.

**RESULTS:**

Two hundred forty-three individuals (36.5%) completed the full survey. LMIC participants engaged in more diverse screening activities and had fewer years of experience (13.5% with more than 20 years of experience *v* 31%; *P* = .048) in screening and were more interested in cervical cancer (76.9% *v* 52.6%; *P* = .002) than HIC participants. However, both groups spent most of their time on research (30.8% LMIC *v* 36.6% HIC; *P* = .518) and agreed that the ICSN biennial meeting enabled them to learn from the experiences of both higher-resource (88.2% *v* 75.7%; *P* = .122) and lower-resource (61.8% *v* 68.0%; *P* = .507) settings. ICSN helped them form new collaborations for research and implementation (55.1% *v* 58.2%; *P* = .063); informed advances in research/evaluation (71.4% *v* 68.0%; *P* = .695), implementation (59.2% *v* 47.9%; *P* = .259), and policies in their settings (55.1% *v* 48.0%; *P* = .425); and provided the opportunity to contribute their knowledge and expertise to assist others (67.3% *v* 71.1%; *P* = .695).

**CONCLUSION:**

Findings suggest that HIC and LMIC participants benefit from knowledge sharing at ICSN meetings although their interests, backgrounds, and needs differ. This points to the importance of international research networks that are inclusive of HIC and LMIC participants in cancer control to advance knowledge and effective practices globally.

## INTRODUCTION

By 2025, the global burden of cancer is expected to exceed 20 million new cancer cases per year with severe consequences on the health and health systems of low- and middle-income countries (LMICs).^1-6^ High-income countries (HICs) have shown some success in controlling the burden of certain cancers through a multipronged approach that includes prevention, diagnosis, and treatment.^2,7^ Cancer screening is a necessary part of this process but ensuring adequate resources and infrastructure to detect, diagnose, and treat cancer while constantly collecting data to evaluate and improve the program is costly.^8,9^ Therefore, decision makers and program managers should use the best evidence available to tailor screening to any given setting and to carefully assess the impact of the program on the burden of cancer at the population level. Successful knowledge sharing of evidence and experiences may inform this decision-making process while moving the science of cancer screening forward.

**CONTEXT**

**Key Objective**
Understand the context of knowledge sharing between high-income country (HIC) and low- and middle-income country (LMIC) participants in the International Cancer Screening Network.
**Knowledge Generated**
Although interests, backgrounds, and needs of HIC and LMIC participants differ, the knowledge available within the International Cancer Screening Network is beneficial to all and there are opportunities for knowledge sharing to happen.
**Relevance**
Interaction of HIC and LMIC participants in international research networks is key to advancing knowledge and effective practices in cancer screening globally.

For the past 30 years, the International Cancer Screening Network (ICSN), led by the National Cancer Institute of the US National Institutes of Health, has facilitated knowledge sharing and research collaboration in the field of cancer screening via a biennial scientific meeting and scientific working groups. ICSN scientific working groups facilitate cross-national research collaboration to assess quality and compare outcomes of cancer screening programs from around the world, contributing to the evidence-base for effective practices in cancer screening.^10-14^

Participants from HICs in North America, Europe, and Asia established the ICSN in the late 1980s as an opportunity to learn from each other’s research and experiences as early implementers of screening mammography. These countries faced a high burden of the disease and were among the first to implement breast cancer screening programs.^10^ In the early 2000s, the ICSN expanded its scope to include cervical and colorectal cancers.^11,12^ In the last few years, as the burden of cancer has grown in LMICs, a number of LMICs have begun to plan and implement screening programs.^15,16^ For example, in 2016, the Government of India developed an Operational Framework for the Management of Common Cancers, which included the design and implementation of breast, cervical, and oral cancer screening.^16^

Now, the ICSN includes LMIC participants who share the latest research evidence on effective design, implementation, and assessment of their cancer screening programs during the ICSN Biennial Meeting. These biennial meetings are open to anyone interested in cancer screening and cover a variety of themes pertinent to the field, such as the latest research evidence on effectiveness of screening programs, screening implementation, impact of screening policies, informed and shared decision making, and others (https://icsn2019.com/). However, cancer screening professionals working in LMICs often do not encounter the necessary resources to develop, implement, and evaluate cancer screening programs.^4,17,18^ One potential benefit of knowledge sharing among ICSN participants based in HICs and LMICs is that it may speed implementation and expansion of effective screening in LMICs by applying available evidence from longstanding successful screening programs based in HICs. On the other hand, HICs could benefit from so-called reverse or frugal innovation,^4,19,20^ learning from screening approaches implemented in LMICs, such as human papilloma virus self-sampling in cervical cancer screening^21,22^ and other novel screening methods that would not be feasible in HICs because of high costs or established standards of care.

The perceptions of stakeholders participating in bidirectional knowledge sharing among HICs and LMICs need additional exploration. The purpose of this analysis was to assess perceptions and experiences of HIC- and LMIC-based participants in the ICSN, with attention to their experiences of bidirectional learning in the context of the ICSN.

## METHODS

Data collection consisted of a survey of ICSN participants and stakeholders from LMICs and HICs. In 2018, the National Cancer Institute fielded a self-administered, online survey to 665 individuals, including 600 from HICs and 65 from LMICs, who had participated in one or both 2015 and 2017 ICSN biennial meetings and/or who had subscribed to a pre-existing ICSN listserv. The survey content was developed on the basis of findings from prior semistructured one-on-one interviews with 14 longstanding participants in the ICSN, who are also globally recognized leaders in the field of cancer screening research. More information about the survey development is available elsewhere.^23^

The 43-question survey instrument consisted of a first part that assessed respondents’ backgrounds and experiences in the field of cancer screening, as well as their engagement with ICSN, and a second part that included questions soliciting feedback on the most recent ICSN biennial meeting, recommendations for the content and structure of future ICSN biennial meetings, perspectives on the value of the ICSN, and degree of interest in proposed future ICSN activities. The full survey instrument is available in the Data Supplement. As an incentive to participate, all individuals who completed the full survey instrument were invited to participate in a drawing to receive one of five US$100 gift cards.

To classify respondents as coming from an HIC or an LMIC, respondents were asked to name the country where they primarily worked. Countries were then coded as HICs or LMICs on the basis of the Atlas method of income classification defined by the World Bank.^24^ The complete list of countries represented in this survey and in the ICSN listserv is available in the Data Supplement. The survey used 4-point Likert scales to measure responses about the extent to which the 2017 ICSN Biennial Meeting enabled participants to learn from different settings, the extent to which ICSN contributed to advance participants’ knowledge and career outcomes, and the extent to which ICSN facilitated knowledge sharing and networking. We categorized responses “1” and “2” into “Disagree” and “3” and “4” into “Agree.”

Quantitative survey data reported herein were analyzed in SAS. Data analysis compared responses from survey participants working in HICs and LMICs through χ^2^ tests. A significance level of .05 was used for all statistical tests.

The US National Institutes of Health Office of Human Subjects Research Protections approved this study.

## RESULTS

A total of 265 individuals completed the first section of the survey (39.8% response rate), and 243 individuals completed the full survey (36.5% response rate). The response rate for the full survey was 75.4% among LMIC participants and 32.3% among HIC participants. Most respondents worked primarily in HICs (Table 1). HIC respondents had more years of experience in the field of cancer screening compared with LMIC respondents (*P* = .048) and were more interested in colorectal (*P* < .001), lung (*P* = .006), and prostate cancers (*P* = .049). LMIC respondents were more interested in cervical cancer (*P* = .002).

**TABLE 1:**
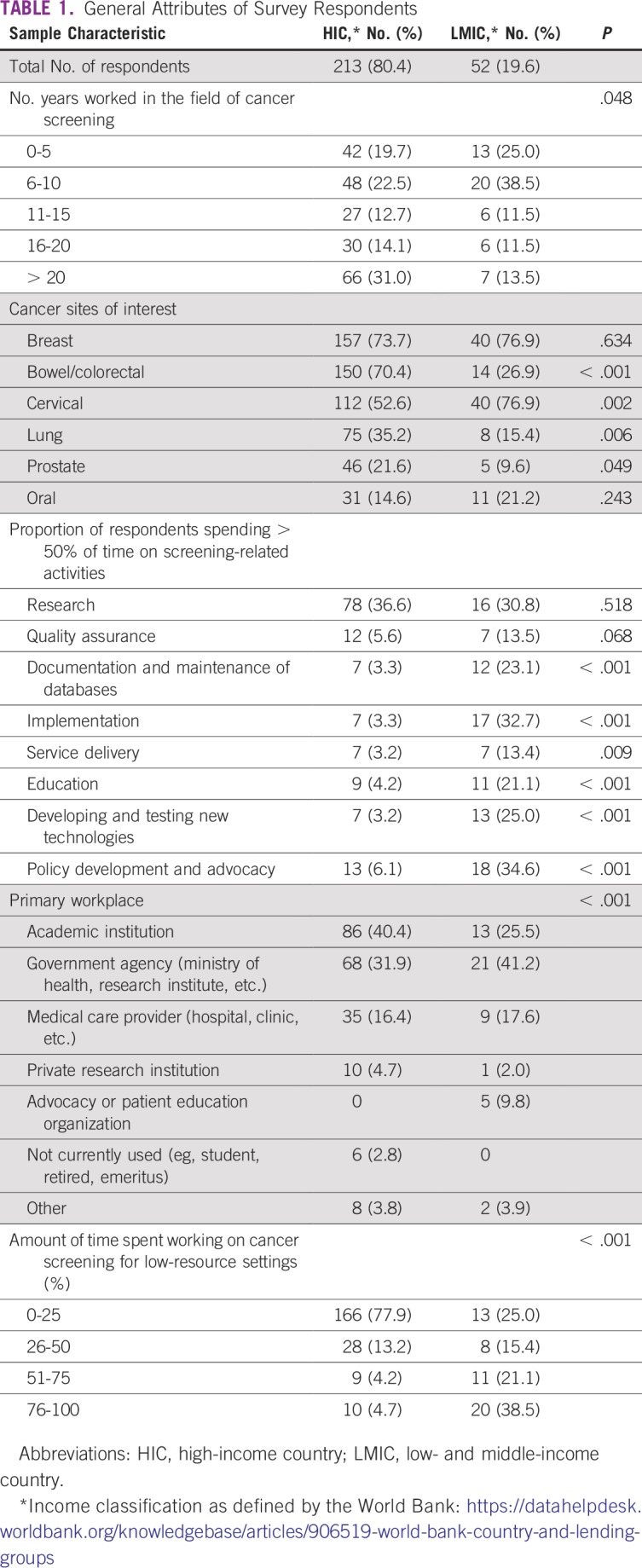
General Attributes of Survey Respondents

More respondents in HICs worked primarily in academic institutions than those in LMICs, while the latter more often identified government agencies as their primary workplace (*P* < .001). Compared with those from HICs, more LMIC respondents spent more than 50% of their time documenting cancer screening activities and maintaining data and databases (*P* < .001), managing cancer screening implementation (*P* < .001), delivering cancer screening clinical services (*P* = .009), educating target populations about screening and cancer (*P* < .001), developing and testing new technologies and tests (*P* < .001), and engaging in policy development or policy advocacy for cancer screening at the subnational or national level (*P* < .001). In addition, more LMIC respondents spent most of their time working in low-resource settings than those in HICs (*P* < .001).

More LMIC than HIC respondents agreed that participating in the ICSN advanced their knowledge of managing cancer screening implementation (*P* = .02). There were no statistically significant differences between HIC and LMIC respondents regarding the extent to which participating in the ICSN advanced their knowledge in conducting research on cancer screening, conducting quality assurance, documenting cancer screening activities, delivering cancer screening services, educating target populations about screening and cancer, developing and testing new screening technologies and tests, and engaging in policy development or policy advocacy for cancer screening at the subnational or national level (Table 2).

**TABLE 2:**
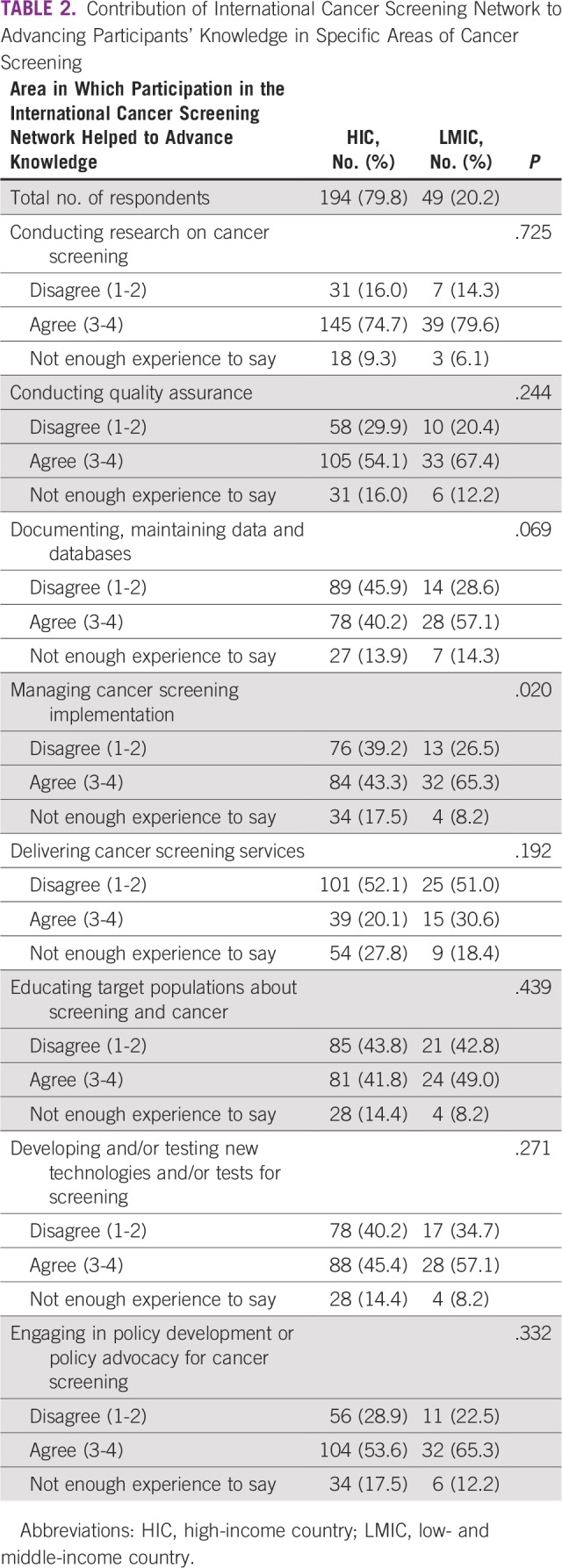
Contribution of International Cancer Screening Network to Advancing Participants’ Knowledge in Specific Areas of Cancer Screening

Most HIC and LMIC respondents agreed that the ICSN facilitates knowledge sharing and networking among individuals working in different cancer screening activities, cancer types, resource settings, and continents (Table 3). Among those who participated in the 2017 ICSN Biennial Meeting, both HIC and LMIC respondents agreed that the biennial meeting enabled them to learn from the experience of cancer screening in higher-resource settings (Table 4). Similarly, most HIC and LMIC respondents agreed that the 2017 ICSN Biennial Meeting enabled them to learn from cancer screening in lower-resource settings.

**TABLE 3:**
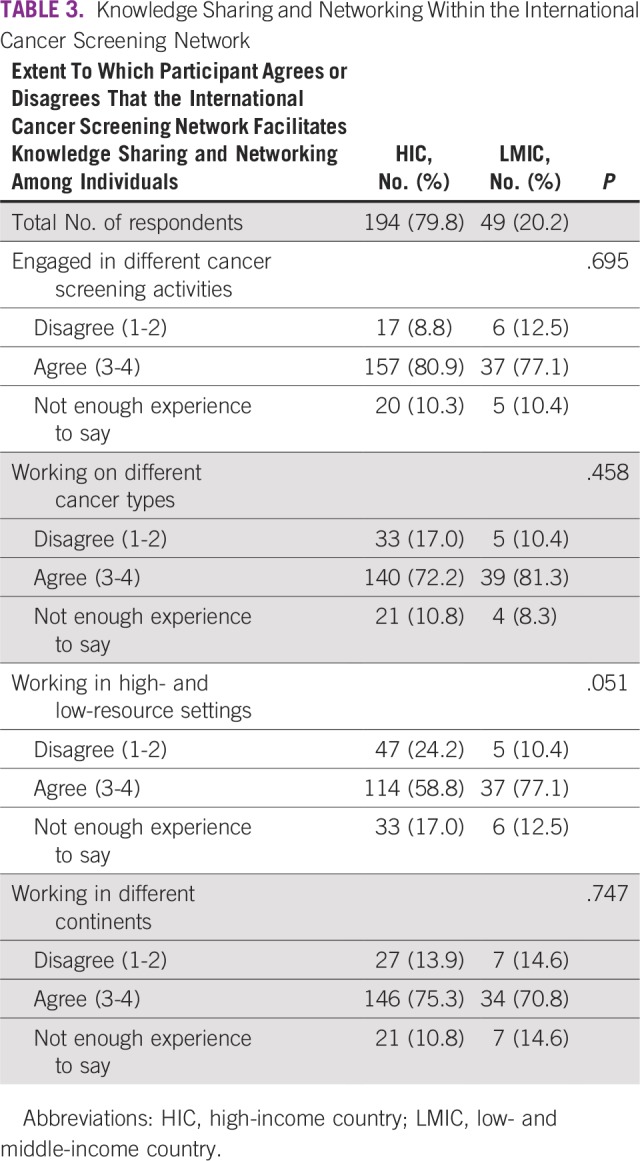
Knowledge Sharing and Networking Within the International Cancer Screening Network

**TABLE 4:**
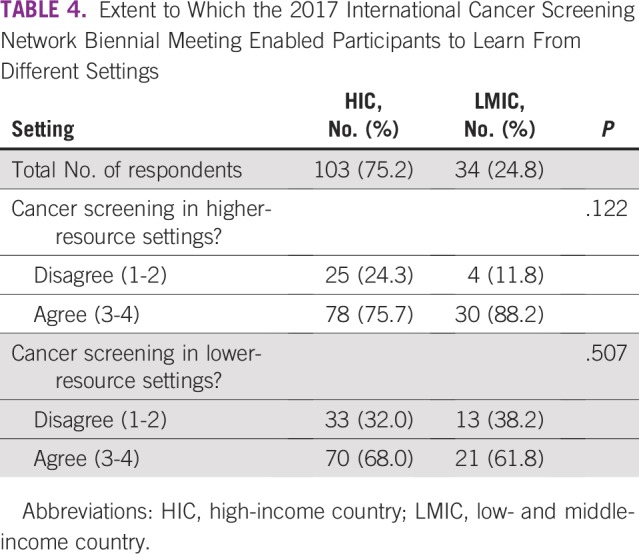
Extent to Which the 2017 International Cancer Screening Network Biennial Meeting Enabled Participants to Learn From Different Settings

In addition, most HIC and LMIC respondents agreed that participating in the ICSN helped them form new collaborations for research and implementation; informed advances in cancer screening research or evaluation, implementation, and policies in their settings; and allowed them the opportunity to share their knowledge and expertise with others (Table 5). Differences between the groups of respondents emerged in response to questions about the ICSN’s contributions to advancing career development and to securing technical assistance for cancer screening implementation. More HIC respondents disagreed that participating in the ICSN contributed to advancing their career development than those in LMICs, while more LMIC respondents indicated that this outcome did not apply to their work (*P* = .007). Regarding technical assistance, more LMIC respondents agreed that participating in the ICSN helped them secure technical assistance for implementation of cancer screening in their setting (*P* < .001).

**TABLE 5:**
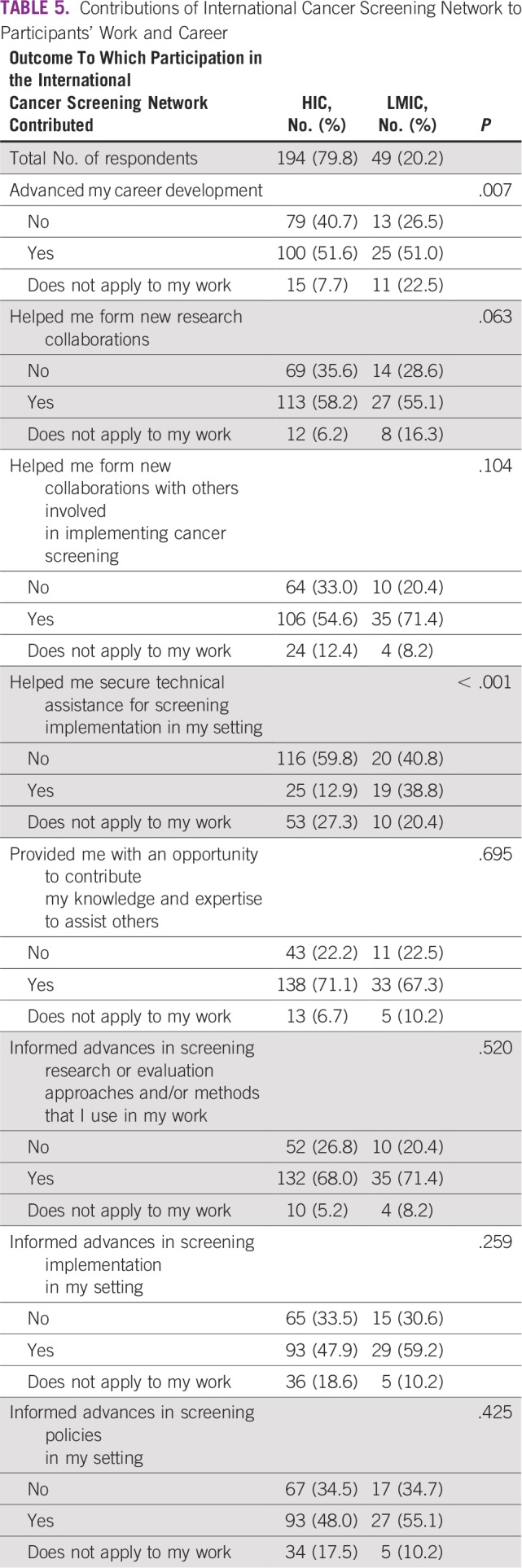
Contributions of International Cancer Screening Network to Participants’ Work and Career

## DISCUSSION

To achieve efficient and effective screening implementation, it is essential that health care professionals and researchers working in HICs and LMICs have access to the best evidence available regarding cancer screening practices. In the past 30 years, ICSN participants have shared their knowledge and worked together through collaborative working groups and the ICSN Biennial Meeting. During this same period, cancer screening programs have made great progress in controlling the burden of certain types of cancer, leading to better prognosis for patients, especially in HICs and in some LMICs.^25,26^ Yet questions exist about the degree to which participants in a global research network working on cancer screening in the different contexts of HICs and LMICs can learn from one another’s research. To learn more about this topic, we analyzed similarities and differences in the interests, needs, experiences, and perceptions of ICSN participants working in HICs and LMICs.

This survey of ICSN participants suggests that cancer screening professionals based in HICs and LMICs have reaped important benefits and identify multiple sources of value from their participation in the ICSN, regardless of the income level of the country where they work. Both groups recognize that participation in the ICSN is beneficial to advancing their knowledge; facilitating knowledge sharing, networking, and collaboration; informing advances in their own screening research and evaluation approaches and/or methods; and informing advances in screening implementation and screening policies in their settings. Moreover, results suggest that knowledge exchange is taking place, because participants from HICs and LMICs reported that the 2017 ICSN Biennial Meeting enabled them to learn from cancer screening in both higher-and lower-resource settings.

However, we found important differences between HIC and LMIC respondents’ characteristics. LMIC respondents had fewer years of experience in the field and were more interested in cervical cancer compared with HIC respondents, who were more interested in lung, colorectal, and prostate cancers. Differences in the cancer burden between HICs and LMICs and consequent priority setting could explain the diverse interests.^27^ For example, the strong interest of LMIC respondents in cervical cancer is in line with the high burden of this disease in those countries. Moreover, cervical cancer is a high priority for global cancer control, with the WHO recently calling for the elimination of cervical cancer worldwide.^28,29^

More LMIC respondents worked in government agencies and were responsible for several cancer screening–related activities (including policy making, implementation, and education), although one-third of them spent most of their time doing research. Those from HICs were usually placed in academic institutions and focused their time on cancer screening research. In addition, there were important differences regarding cancer screening implementation—more LMIC respondents agreed that the meeting advanced their knowledge on how to manage cancer screening implementation and helped them secure technical assistance for it.

When assessing resource availability and the implementation of cancer screening programs at regional or national levels, it is important to consider that countries with heterogeneous populations and vast territories often present areas with fewer human and financial resources and more limited infrastructure. These are often characterized as lower-resource settings, occurring in both HICs and LMICs, and they face similar challenges in terms of health care delivery.^30^ For example, women living in US Appalachia present a burden of cervical cancer similar to that of many LMICs and higher than the US overall rate.^31^ Conversely, some settings within LMICs may present a cancer burden and availability of health resources closer to those typically found in HICs. For example, the Brazilian state of São Paulo produces one-third of the country’s gross domestic product and experiences breast cancer incidence and mortality at 57.5 cases and 17.2 deaths per 100,000 women^32^ compared with an average of 78.3 cases and 12.9 deaths per 100,000 women reported in HICs overall.^27^

However, we did not define “higher-resource” and “lower-resource” settings in the survey. These terms are not well defined in the literature and are often conflated with HICs and LMICs,^30,33^ which may have led some respondents to improperly identify them. In addition, because of the exploratory nature of this study and its small sample size, we did not correct for multiple comparisons in the analysis, and some of the reported differences may be the result of chance. In future research on bidirectional learning among HIC and LMIC participants, it will be important to offer adequate definitions of these settings and to parse out their influence within a randomly assigned sample of participants.

Although the survey sample is limited to ICSN participants, it is important to note that the ICSN and its Biennial Meetings offer a unique setting for knowledge exchange in the field and attract cancer screening researchers, evaluators, and implementers, in early or late-stage careers, from around the world with interests in diverse areas. The different response rates between HIC and LMIC participants may have affected our results. If we assume that those answering the survey were more willing to offer positive feedback in the areas of knowledge sharing and benefits of their participation in the ICSN, we could then expect that our respondents were more likely to agree regarding the positive aspects of the network and discord may be obscured by the silence of dissatisfied nonrespondents. Because the network is a voluntary organization, we are reassured that the needs of those who are engaged and responding are being served.

Overall, the similarities and differences between HIC and LMIC participants can together inform thinking about bidirectional knowledge exchange among these groups. However, important questions remain regarding knowledge sharing among LMICs and HICs in cancer screening. We need to investigate how best to leverage the lessons learned from decades of research, implementation, and evaluation of cancer screening programs in HICs to benefit screening in LMICs, given the range of social, cultural, political, economic, and health system differences among HICs and LMICs, and among countries within each of these groups. Although the transfer of knowledge, such as guidelines and screening strategies, normally moves from HICs to LMICs,^15,34,35^ it is possible to imagine that strategies developed in LMICs may be adapted to low-resource settings in HICs, allowing them to optimize their resources to improve their programs and delivery of care. For instance, human papilloma virus self-sampling in cervical cancer screening, often evaluated in LMICs such as China and Haiti, could also be a successful strategy to reach underserved women in low-resource settings in HICs.^36^

The experiences of cancer screening professionals in HICs could inform the work of professionals who are newly establishing cancer screening programs (and related research and evaluation activities) in LMICs. Moreover, the challenges of cancer screening implementation in LMICs may provide opportunities for early-career professionals to apply and develop their skills, while investigating research questions relevant to the field. Considering that research is the common language and a primary benefit to all ICSN participants, these research opportunities could frame the work of the ICSN going forward and cancer screening professionals working in HICs and LMICs would have much to gain from them.

In conclusion, the increasing burden of cancer in LMICs is a serious challenge that must be addressed with effective strategies for prevention and control, including cancer screening, that are appropriately adapted to lower-resource settings. The ICSN provides an opportunity for knowledge sharing between cancer screening professionals working in HICs and LMICs about the latest evidence for effective practices in cancer screening. This bidirectional learning may help LMICs to develop research strategies, avoid recognized mistakes, and save valuable resources in the development and implementation of cancer screening. It may also help HICs to adapt approaches developed in LMICs. Findings from this study point to the value of knowledge sharing for both groups of participants in the ICSN. Findings can be extrapolated to the importance of international research networks that are inclusive of HIC and LMIC participants in cancer control, as well as noncommunicable disease control more broadly, to advancing knowledge and effective practices globally.
